# Carbon Monoxide Is Involved in Hydrogen Gas-Induced Adventitious Root Development in Cucumber under Simulated Drought Stress

**DOI:** 10.3389/fpls.2017.00128

**Published:** 2017-02-07

**Authors:** Yue Chen, Meng Wang, Linli Hu, Weibiao Liao, Mohammed M. Dawuda, Chunlan Li

**Affiliations:** College of Horticulture, Gansu Agricultural UniversityLanzhou, China

**Keywords:** adventitious rooting, drought, HRW, CO, chlorophyll fluorescence, enzyme activity

## Abstract

Hydrogen gas (H_2_) and carbon monoxide (CO) are involved in plant growth and developmental processes and may induce plant tolerance to several stresses. However, the independent roles and interaction effect of H_2_ and CO in adventitious root development under drought conditions have still not received the needed research attention. We hypothesize that there exists crosstalk between H_2_ and CO during adventitious root development under drought stress. The results of our current study revealed that 50% (v/v) hydrogen-rich water (HRW), 500 μM Hemin (the CO donor) and 30% (w/v) CO aqueous solution apparently promoted the development of adventitious roots in cucumber explants (*Cucumis Sativus* L.) under drought stress. H_2_ and CO increased relative water content (RWC), leaf chlorophyll content (chlorophyll a, b, and a+b), and chlorophyll fluorescence parameters [photochemical efficiency of photosystem II (PSII), PSII actual photochemical efficiency and photochemical quench coefficient] under drought condition. When the CO scavenger hemoglobin (Hb) or zinc protoporphyrin IX (ZnPPIX) was added to HRW/CO aqueous solution, the positive effect of HRW/CO aqueous solution on RWC, leaf chlorophyll content, and chlorophyll fluorescence parameters were reversed. Additionally, superoxide dismutases, peroxidase, catalase, and ascorbate peroxidase was significantly increased in the explants treated with HRW and CO aqueous solution under drought stress, thus alleviating oxidative damage, as indicated by decreases in thiobarbituric acid reactive substances (TBARS), hydrogen peroxide (H_2_O_2_), and superoxide radical (O_2_^-^) levels. H_2_ and CO also improved the levels of water soluble carbohydrate, total soluble protein, and proline content. However, the above CO/H_2_-mediated effects were reversed by CO scavenger Hb or CO specific synthetic inhibitor ZnPPIX. Therefore, CO may be involved in H_2_-induced adventitious rooting under drought stress and alleviate oxidative damage by enhancing RWC, leaf chlorophyll content, chlorophyll fluorescence parameters, metabolic constituent content, activating anti-oxidant enzymes and reducing TBARS, O_2_^-^, and H_2_O_2_ levels.

## Introduction

Drought stress which is among the important abiotic stresses that adversely affects plant growth and development has become a global problem (Farooq et al., 2009). In order to withstand drought, plants develop multiple defense mechanisms. Recently, many studies have focused on understanding adventitious rooting response signaling pathways under drought stress. Adventitious root formation is a very complex process. Enzymes such as peroxidase (POD), polyphenol oxidase (PPO), and indoleacetic acid oxidase (IAAO) are known to be intimately involved in the process of adventitious root formation (Smart et al., 2003). This process is affected by factors such as temperature, light conditions, water, and nutrient supply (Takahashi et al., 2003). Among plant hormones, auxins are of vital importance, because they can promote the formation of primordial roots. Recent studies found signal molecules that regulate in auxin-induced adventitious root development, for instance hydrogen peroxide (H_2_O_2_, Liao et al., 2012; Niu and Liao, 2016), nitric oxide (NO, Zhu et al., 2016b), carbon monoxide (CO, Wang and Liao, 2016), hydrogen sulfide (H_2_S, Lin et al., 2012), and calcium (Ca^2+^, Cui et al., 2015). To date, however, the intricate signaling network that participates in adventitious root development remains unresolved.

Hydrogen gas (H_2_) is colorless, odorless, and tasteless, and is considered to be physiologically inert molecule. H_2_ was considered to be a selective reductant (Ohsawa et al., 2007) and be involved in the development and stress responses in plants (Renwick et al., 1964; Zeng et al., 2013). Hydrogen-rich water (HRW) treatment could delay postharvest ripening and senescence of kiwifruit by reducing the rot incidence, inhibiting the respiration intensity, and decreasing the lipid perxidation level postharvest fruits (Hu et al., 2014). H_2_ enhanced *Arabidopsis* salt tolerance by modulating genes/proteins of zinc-finger transcription factor ZAT10/12 (Xie et al., 2012). Exogenous HRW effectively led to an increase of intracellular H_2_ production, and a reduction in the stomatal aperture, and therefore resulted in enhanced drought tolerance (Xie et al., 2014). Wu et al. (2015) found that 50% HRW significantly alleviated the cadmium toxic symptoms by increasing the antioxidant capacities in Chinese cabbage. HRW conferred tolerance to UVB-induced oxidative damage partially by the manipulation of (iso)flavonoids metabolism in *Medicago sativa* L. (Xie et al., 2015). HRW significantly blocked UV-A-induced the accumulation of H_2_O_2_ and O_2_ (Su et al., 2014). Results of previous studies have shown that H_2_ promoted adventitious root development in a CO-dependent manner (Lin et al., 2014). During adventitious rooting, H_2_ mediated target genes related to auxin signaling and adventitious root development, *CsDNAJ-1, CsCPDK1/5, CsCDC6, CsAUX228*-like, and *CsAUX22D*-like through CO pathways. Our results also confirmed that H_2_ promoted adventitious rooting by increased the content of NO and the activities of NO synthase and nitrate reductase (Zhu et al., 2016b). Additionally, H_2_ activated cell cycle and up-regulated cell cycle-related genes and adventitious rooting-related genes via NO pathway (Zhu et al., 2016a).

Carbon monoxide is one of the most important reactive trace gases in nature, which has long been widely considered as a poisonous gas. Siegel et al. (1962) reported that germinating seeds of rye (*Secale cereale*), pea (*Pisum sativum*), cucumber, and lettuce (*Lactuca sativa*) produced CO at levels of 10 to 25 mL L^-1^. Piantadosi (2002) found that CO influenced cell proliferation and the production of cytokines in plants. CO is bioactive molecule involved in many biological processes. It has been reported that CO played a major role in mediating the cytoprotection against oxidant-induced lung injury and neurotransmission (Huang et al., 2006). In addition, CO could acts as a signal molecule involved in plant growth and development which requires the participation of other signaling molecules. For instance, CO delayed gibberellins (GA)-triggered programmed cell death (PCD) in wheat aleurone cells by up-regulating of ascorbate peroxidase (APX) and catalase (CAT) expression, and decreasing H_2_O_2_ overproduction (Wu et al., 2010). Low CO concentrations (0.1 or 1%) in air stimulated seed germination in dormant giant foxtail (*Setaria faberi*; Dekker and Hargrove, 2002). CO produced by heme oxygenase might be involved in ABA-induced stomatal closure in *Vicia faba* (Cao et al., 2007). CO enhanced antioxidant defense system in abiotic stresses tolerance, such as drought (Liu et al., 2010), salt (Ling et al., 2009), ultraviolet radiation (Yannarelli et al., 2006), and heavy metal (Meng et al., 2011). Xuan et al. (2008) found that CO induced adventitious rooting in dose- and time-dependent manners. The application of CO aqueous solution was able to alleviate the IAA depletion-induced inhibition of adventitious rooting. Methane-rich water stimulated adventitious rooting, which was partially mediated by CO in cucumber (Cui et al., 2015). Moreover, CO showed cross talk with other signaling molecules including NO, phytohormones (IAA, ABA, and GA), H_2_S, H_2_, and CH_4_ (Wang and Liao, 2016).

H_2_ and CO as new gas signaling modulators are involved in signal path ways of plants. Studies have shown the involvement of heme oxygenase-1 (HO-1, a novel antioxidant enzyme)/CO in H_2_-induced osmotic stress tolerance in alfalfa (Jin et al., 2016). In addition, H_2_ mediated target genes related to auxin signaling and adventitious root development through CO pathways during adventitious rooting (Lin et al., 2014). As mentioned above, both H_2_ and CO could act as important gaseous molecules with multiple biological functions in plant response against abiotic stress. However, the signal roles of H_2_ and CO under abiotic stress during adventitious rooting remain unclear. It may be hypothesized that H_2_, CO, and their crosstalk could be involved in adventitious root development in plants under drought stresses. Therefore, the present study focuses on the effect of H_2_ and CO on the adventitious root development in cucumber under drought stress and their relationship during that process. The results presented in this work are significant for both fundamental and applied plant biology.

## Materials and Methods

### Plant Materials

Cucumber seeds (*Cucumis Sativus* ‘Xinchun No. 4’) were purchased from the Gansu Academy of Agricultural Sciences in Lanzhou, China. The seeds were surface sterilized in 5% (w/v) sodium hypochlorite for 10 min and then soaked in distilled water for 5 h. The seeds were germinated on filter paper with distilled water in Petri dishes (15 cm-diameter, 2.5 cm-deep) and maintained at 25 ± 1°C for 7 days with a 14-h photoperiod (photosynthetically active radiation = 200 μmol m^-2^ s^-1^). The 7-day-old cucumber seedlings with primary roots removed were used as explants and maintained under the same temperature and photoperiod conditions for another 7 days in the presence of different media as indicated below. The number of adventitious roots per explant was counted and recorded.

### Experimental Treatments

**FIGURE 1:**
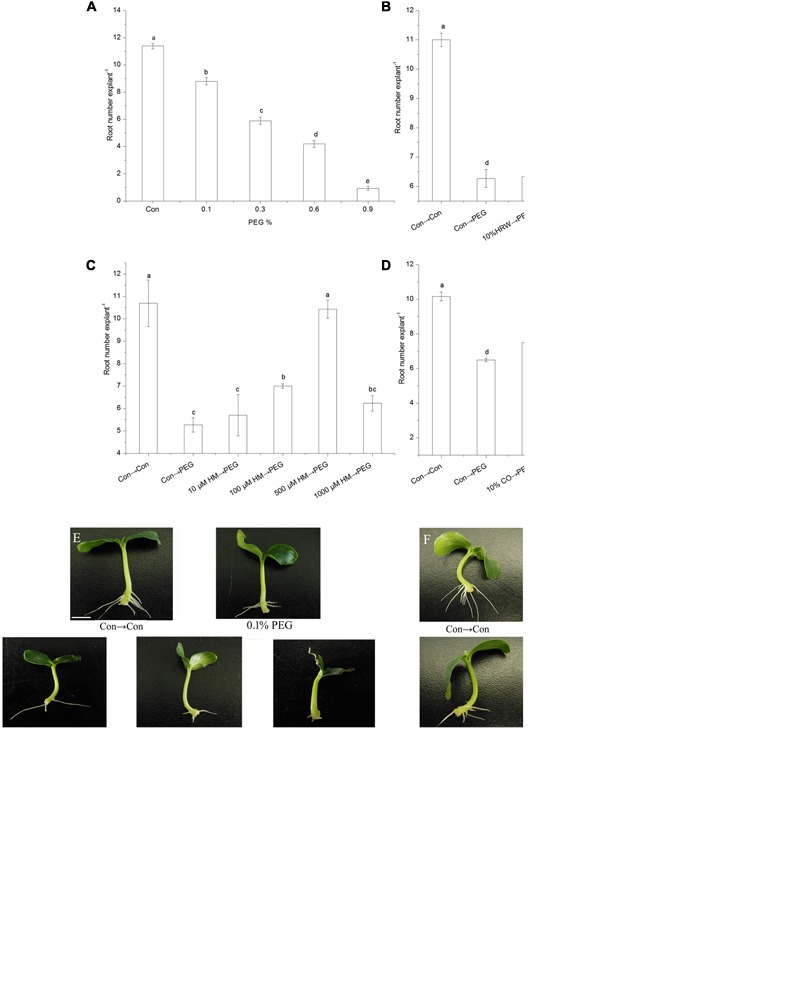
**Effects of different concentrations of exogenous H_2_ and CO on adventitious root in cucumber under drought stress.** The primary root was removed from hypocotyl of 7-day-old germinated cucumber. Explants were incubated in the solution containing different concentrations of PEG **(A,E)**, subsequently the number of adventitious root were measured after 7 days. Explants were pretreated with or without different concentrations of HRW **(B,F)**, Hemin **(C,G)**, or CO aqueous solution **(D,H)** for 48 h, and then transferred on agar plate containing 0 or 0.3% (w/v) PEG. Explants without PEG treatments were used as the control (Con→Con). The number of adventitious root were measured after 5 days. Means and SE values were calculated from at least three independent experiment (*n* = 3 with 10 explants per replication). Bars denoted by the same letter indicate no significant difference at *p* < 0.05 according to Duncan’s multiple range tests. Bar = 1 cm.

All the chemicals used in the experiments were obtained from Sigma (St. Louis, MO, USA) unless otherwise stated. Drought stress was simulated by application of polyethylene glycol 6000 (PEG; obtained from Shanghai Chemical Reagent Co. Ltd. Shanghai, China). Based on previous reports, we used different concentrations of PEG (0.1, 0.3, 0.6, and 0.9%, m/v), HRW (10, 30, and 50%, v/v; Xie et al., 2014), the CO donor Hemin (10, 100, 500, and 1000 μM) and CO aqueous solution (10, 30, and 50%, w/v; Xuan et al., 2008) as indicated in **Figure 1** and kept at 25 ± 1°C. The compound zinc protoporphyrin IX (ZnPPIX, Sigma) was used at 100 μmol L^-1^ as a specific inhibitor of CO. Hemoglobin (Hb, Sigma) was chosen as CO scavenger at 0.5 g L^-1^ (Xuan et al., 2008). Seven days old explants which were treated distilled water throughout the experiment served as control to provide a basis to compare the effects of H_2_ and CO under drought conditions. The experiment included nine treatments: (1) Con→Con (the control; Con) explants treated with distilled water for 7 days. (2) Con→PEG (PEG treatment), explants pretreated with distilled water for 2 days and then transferred to PEG solution for 5 days. (3) HRW→PEG (HRW treatment), explants pretreated with HRW for 2 days and then transferred to PEG solution for 5 days. (4) CO→PEG (CO treatment), explants pretreated with CO aqueous solution for 2 days and then transferred to PEG solution for 5 days. (5) CO+HRW→PEG (CO+HRW treatment), explants pretreated with CO+HRW for 2 days and then transferred to PEG solution for 5 days. (6) HRW+Hb→PEG (HRW+Hb treatment), explants pretreated with HRW+Hb for 2 days and then transferred to PEG solution for 5 days. (7) HRW+ZnPPIX→PEG (HRW+ZnPPIX treatment), explants pretreated with HRW+ZnPPIX for 2 days and then transferred to PEG solution for 5 days. (8) CO+Hb→PEG (CO+Hb treatment), explants pretreated with CO+Hb for 2 days and then transferred to PEG solution for 5 days. (9) CO+ZnPPIX→PEG (CO+ZnPPIX treatment), explants pretreated with CO+ZnPPIX for 2 days and then transferred to PEG solution for 5 days. The concentration of these chemicals was selected based on the results of a preliminary experiment. The treatments were arranged in a completely randomized design with at least three replications.

### Leaf Relative Water Content

Leaf relative water content (RWC) was measured as described by Aroca et al. (2003) after 0, 12, 24, and 48 h of PEG treatment. One leaf samples per explants were immediately weighed (fresh weight) then placed in a water vapor-saturated vial for 24 h and weighed (turgid weight). The samples were dried in an oven at 80°C for 48 h and their dry weights (dry weight) were determined. At each point in time, three replicates were performed. RWC was calculated by the following equation: [(fresh weight - dry weight)/(turgid weight - dry weight)] × 100.

### Leaf Chlorophyll Content and Leaf Chlorophyll Fluorescence

To determined chlorophyll content, 0.5 g leaves were frozen in liquid nitrogen, grounded to a powder, and extracted in 5 ml of 80% (v/v) acetone until complete bleaching. Chlorophyll was quantified by measuring the absorption at 645 and 663 nm and its concentration was calculated according to Arnon (1949). Chlorophyll content was expressed in mg⋅g^-1^ fresh weight. Chlorophyll fluorescence was measured with modulated chlorophyll fluorescence imaging system Imaging-PAM (Walz, Effeltrich, Germany). Chl fluorescence parameters were calculated using the following equations: maximal quantum yield of photosystem II (PSII), *F*v/*F*m; quantum efficiency of PSII electron transport, ΦPS II = (*F*m′ -*F*s)/*F*m′; photochemical quenching, qP = (*F*m′ -*F*s)/*F*v′. The seedlings leaves were kept in darkness for 30 min to allow all reaction centers to open. Chl content and chl fluorescence were measured after 0, 12, 24, and 48 h of PEG treatment, respectively.

### Determination of TBARS, O_2_^-^, and H_2_O_2_

The level of lipid peroxidation was measured in terms of thiobarbituric acid reactive substances (TBARS) concentration (Heath and Packer, 1968). Fresh leaf samples (0.2 g) were homogenized in 10 mL of 0.1% trichloroacetic acid (TCA). The homogenate was centrifuged at 1699 × *g* for 10 min. About 2 mL of aliquot of the supernatant was mixed with 4 mL of 0.5% TBA in 20% TCA. The mixture was heated at 95°C for 20 min and then quickly cooled in an ice bath. After centrifugation at 10621 × *g* for 10 min to remove the suspended turbidity, the absorbance of the supernatant was recorded at 523, 600, and 450 nm, respectively. The TBARS concentration was calculated using its absorption coefficient (155 mM^-1^cm^-1^).

Superoxide radical (O_2_^-^) level was measured as described by Elstner and Heupel (1976) with some modifications. One gram of frozen leaf segments was homogenized with 3 ml of 65 mM potassium phosphate buffer (pH 7.8) and centrifuged at 2655 × *g* for 10 min. The incubation mixture contained 0.9 ml of 65 mM phosphate buffer (pH 7.8), 0.1 ml of 10 mM hydroxylamine hydrochloride, and 1 ml of the supernatant. After incubation at 25°C for 20 min, 17 mM sulfanilamide and 7 mM-naphthylamine were added to the incubation mixture. After reaction at 25°C for 20 min, ethyl ether in the same volume was added and centrifuged at 1699 × *g* for 5 min. The content of O_2_^-^ was estimated by measuring the spectrum absorbance of the supernatant at 530 nm and using a standard curve plotted with a known concentration of NO_2_^-^.

H_2_O_2_ content in cucumber explants was determined according to Mukherjee and Choudhuri (1983) with some modifications. Cucumber explants (0.2 g) were homogenized in an ice bath with 2 mL of 0.1% (w/v) TCA. The homogenate was centrifuged at 1699 × *g* for 10 min and 0.5 mL of the supernatant was added to 0.5 mL of 10 mM potassium phosphate buffer (pH7.0) and 1 mL of 1 M KI. The content of H_2_O_2_ was estimated by measuring the spectrum absorbance of the supernatant at 415 nm and using a standard curve plotted with a known concentration of H_2_O_2_.

### Antioxidant Enzymes Assays

The supernatants used for assays of enzyme activity were prepared as described by Jin et al. (2013) with some modifications. Total superoxide dismutases (SOD) activity was assayed by measuring its capacity of inhibiting the photochemical reduction of Nitrotetrazolium Blue chloride (NBT). One unit of SOD (U) was defined as the amount of crude enzyme extract required to inhibit the reduction rate of NBT by 50%. POD was determined by measuring the oxidation of guaiacol (extinction coefficient 26.6 mM^-1^ cm^-1^) at 470 nm. CAT activity was determined by following the consumption of H_2_O_2_ (extinction coefficient 39.4 mM^-1^cm^-1^) at 240 nm for 3 min. The reaction mixture contained 0.025 mol L^-1^ potassium phosphate buffer (pH 7.0), 20 mM H_2_O_2_ and 0.1 ml of enzyme extract in a 3 ml volume. APX activity was determined by monitoring the decrease at 290 nm (extinction coefficient 2.8 mM^-1^cm^-1^).

### Osmotic Regulators Content Estimation

Water soluble carbohydrate was determined after 0, 12, 24, and 48 h of PEG treatment using the anthrone method as described by Liao et al. (2012) with some modifications. Water soluble carbohydrate was extracted for 12 h at 70°C after the explants samples (0.2 g) had been homogenized. The carbohydrate extract was analyzed by reacting 0.5 mL of the supernatant with 0.5 mL of freshly prepared anthrone reagent (1 g anthrone in 50 mL ethyl acetate), 1.5 mL distilled H_2_O and 5 mL of concentrated sulfuric acid, and then placing it in boiling water bath for 1 min. After cooling to room temperature, the absorbance at 630 nm was measured. Total soluble protein content was measured according to the procedure described by Bradford (1976), using bovine serum albumin as a calibration standard. The soluble protein fraction was obtained after centrifugation of the crude extract at 1699 × *g* and 4°C for 10 min.

Determination of proline content was done according to Bates et al. (1973) with some modifications. Cucumber explants (0.2 g) from each group were homogenized in 3% (w/v) sulphosalicylic acid and homogenate filtered through filter paper. After addition of acid ninhydrin and glacial acetic acid, the resultant mixture was heated at 100°C for 1 h in water bath. Reaction was then stopped by using ice bath. The mixture was extracted with toluene, and the absorbance of fraction with toluene aspired from liquid phase was read at 520 nm.

### Statistical Analysis

Results were expressed as the mean values ± SE of three independent experiments (*n* = 3). In the determination of root number, 10 cuttings were used per replication. In the determination of the physiological and biochemical indicators, we used different weight sample per replication which were mentioned in the assay methods. Statistical analysis was performed using software SPSS 17.0. Analysis of Variance (ANOVA) was done and the Duncan’s multiple range test (*P* < 0.05) was used in separating treatment means which showed significant differences from the analysis of variance conducted.

## Results

### Effect of Exogenous Pretreatments with H_2_ and CO on Adventitious Root Development under Simulated Drought Stress

As shown in **Figure 1**, the number of adventitious root significantly decreased with the increase of PEG concentration. Compared with the control (Con), the root number of 0.1, 0.3, 0.6, and 0.9% PEG treated explants decreased by 22.81, 48.25, 63.16, and 91.84%, respectively. Therefore, treatments with 0.1% PEG, 0.3% PEG, and 0.6–0.9% PEG could be termed as low, moderate, and severe drought stress, respectively. The 0.3% PEG was used to simulate moderate drought in the subsequent experiments.

To study the effects of exogenous H_2_ and CO on adventitious root development under drought stress, we performed dose-response experiments with HRW, the CO donor Hemin and CO aqueous solution. When compared with the control explants, treatment with PEG significantly decreased adventitious root number (**Figure 1**). Explants pretreated with 30, 50, and 100% HRW had a significant increase in root number compared with PEG-treated explants, indicating that PEG-induced drought stress was alleviated by HRW. Among the various HRW concentrations, 50% HRW promoted rooting better than the other treatments (**Figure 1**).

As shown in **Figure 1**, pretreatments with different concentrations of Hemin (100–1000 μM) increased adventitious root number under drought stress compared with PEG treatment. The maximum root number was observed in the 500 μM Hemin treatment, reached the level of the control. **Figure 1** showed that 10 and 30% CO aqueous solution pretreatments resulted in increased root number under drought stress. However, 50 and 100% CO aqueous solution pretreatments decreased root number under drought stress. Therefore, 500 μM Hemin and 30% CO aqueous solution which were the most effective under our experimental conditions were used for further studies.

### Effect of Pretreatments with Hb and ZnPPIX on Adventitious Root Development under Simulated Drought Stress

**FIGURE 2:**
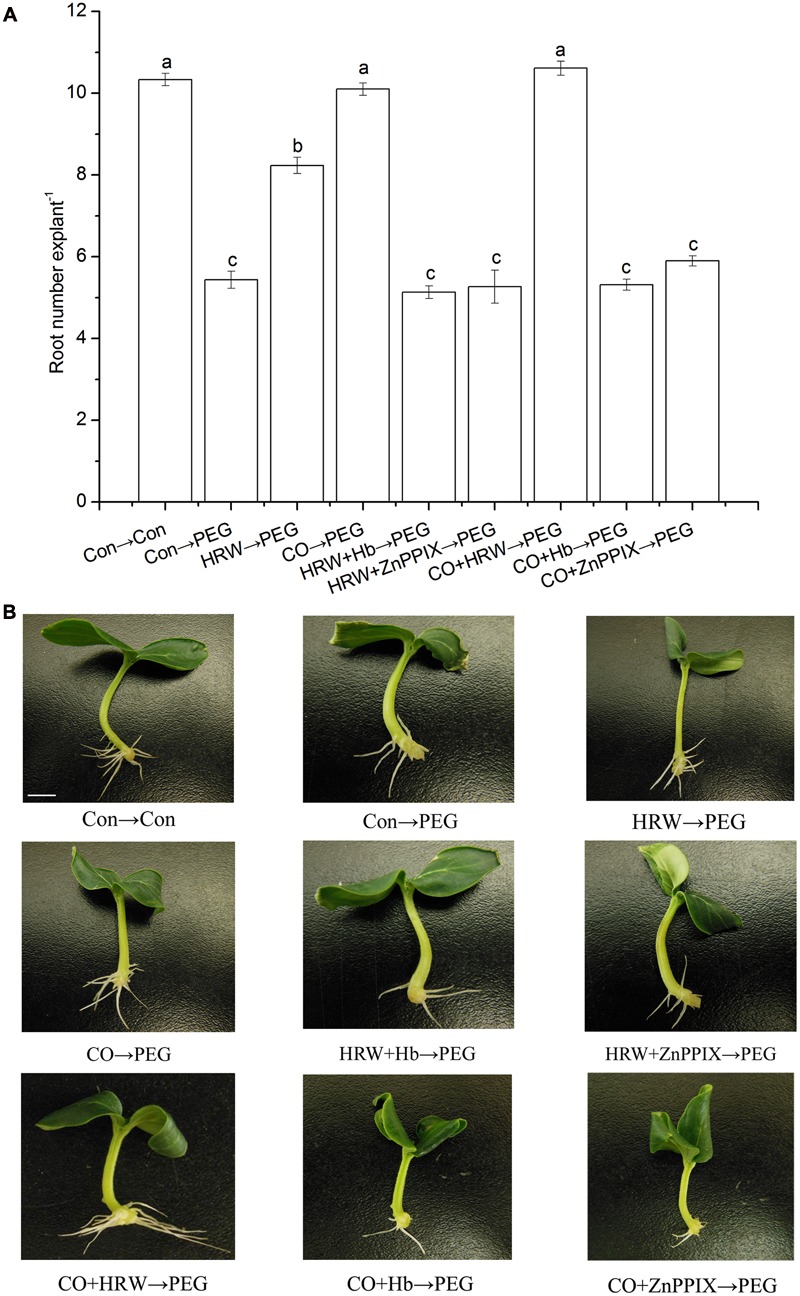
**Effect of CO scavenger Hb or the CO-specific synthetic inhibitor ZnPPIX on adventitious root in cucumber explants under drought stress.** The primary root was removed from hypocotyls 7-day-old germinated cucumbers. Explants were pretreated with or without Hb or ZnPPIX for 48 h, and then transferred on agar plate containing 0 or 0.3% (w/v) PEG. Explants without PEG treatments were used as the control (Con→Con). Means and SE values **(A)** were calculated from at least three independent experiment (*n* = 3 with 10 explants per replication). Bars denoted by the same letter indicate no significant difference at *p* < 0.05 according to Duncan’s multiple range tests. Bar = 1 cm. Photographs **(B)** show hypocotyl explants after 7 days of the treatments indicated.

To understand the interaction effect of H_2_ and CO, we studied the effects of CO scavenger or inhibitor on H_2_-induced adventitious rooting under drought stress. As shown in **Figure 2**, HRW, CO, and CO+HRW increased root number under drought stress. The HRW/CO-induced adventitious rooting under drought condition was reserved by CO scavenger Hb and inhibitor ZnPPIX. Thus, CO might be involved in H_2_-induced adventitious root formation under drought condition.

### Effect of Pretreatments with H_2_ and CO on Leaf Relative Water Content during Adventitious Rooting under Simulated Drought Stress

**FIGURE 3:**
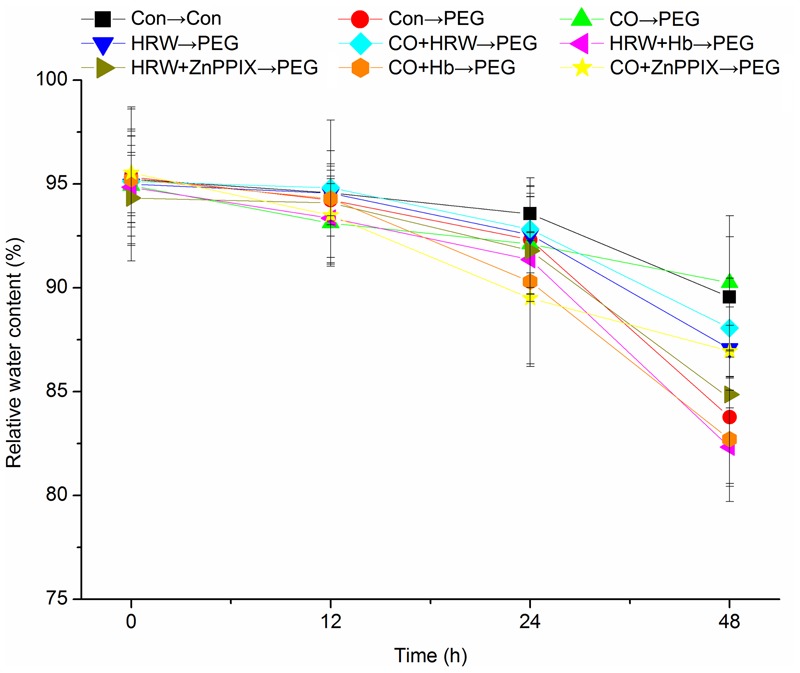
**Effects of H_2_ and CO on the relative water content in cucumber explants leaves under drought stress.** Rooting were Con (square), PEG (circle), CO (regular triangle), HRW (inverted triangle), CO+HRE (diamond), HRW+Hb (left triangle), HRW+ZnPPIX (right triangle), CO+Hb (hexagon), or CO+ZnPPIX (pentagram) as indicated. The concentrations of PEG, HRW, and CO were used at 0.3% (w/v), 50% (v/v), and 500 μM, respectively. Vertical bars represent mean ± SE value from three independent experiments.

Leaf RWC decreased continuously with the progressive drought in all treatments (**Figure 3**). Compared with the control, PEG treatment resulted in an obvious reduction in RWC. The RWC in treatments with HRW, CO, and CO+HRW were significantly higher than that in PEG treatment at 24 and 48 h (*P* < 0.05). Compared with PEG treatment, HRW, CO, and CO+HRW treatments increased RWC by 3.95, 7.72, and 5.12% at 48 h, respectively. When Hb or ZnPPIX was added to HRW/CO, RWC significantly decreased at 24 and 48 h (*P* < 0.05). These results indicate that PEG reduced RWC during adventitious rooting and H_2_ and CO alleviated the reduction. Meanwhile, Hb or ZnPPIX partially reversed the positive effects of H_2_ and CO on RWC.

### Effect of Pretreatments with H_2_ and CO on Leaf Chlorophyll Content and Chlorophyll Fluorescence Parameters during Adventitious Rooting under Simulated Drought Stress

Chlorophyll quantification has been widely used to estimate plant response to various stresses. Therefore, the effects of HRW and CO on leaf chlorophyll content were evaluated during adventitious rooting under drought condition. Compared with control, the contents of chl a and chl a+b decreased at 12, 24, and 48 h in PEG treatment explants (**Table 1**). Compared with PEG, the chl a and chl a+b content in treatments with HRW, CO, and CO+HRW significantly increased during 0–48 h. When Hb and ZnPPIX were added to HRW and CO, the chl a and chl a+b content decreased at 0–48 h. Compared with the control, drought stress (PEG treatment) resulted in a decrease in chl b content at 12, 24, and 48 h. However, a significant increase in the chl b content was observed in the treatments with HRW, CO and CO+HRW during 12–48 h. A significant decrease in chl b in HRW/CO+Hb and HRW/CO+ZnPPIX treatments were observed at 12–48 h, compared with the HRW (**Table 1**). Therefore, H_2_ and CO may maintain chlorophyll content under drought stress during adventitious rooting, and CO might be involved in H_2_-induced chlorophyll content promotion in cucumber.

**Table 1:** Effect of H_2_ and CO on leaf chlorophyll content during adventitious rooting in cucumber explants under drought stress.

Index	Treatments	Time (h)			
		0	12	24	48
Chl a	Con→Con	1.14 ± 0.09c	1.11 ± 0.02a	1.16 ± 0.04a	1.16 ± 0.11a
	Con→PEG	1.14 ± 0.03c	0.91 ± 0.10c	0.79 ± 0.11c	0.41 ± 0.03d
	HRW→PEG	1.18 ± 0.03b	1.09 ± 0.03b	0.93 ± 0.04b	0.93 ± 0.03b
	CO→PEG	1.26 ± 0.04a	1.11 ± 0.02a	1.16 ± 0.03a	1.19 ± 0.03a
	CO+HRW→PEG	1.16 ± 0.05a	1.13 ± 0.02a	1.14 ± 0.01a	1.21 ± 0.08a
	HRW+Hb→PEG	1.09 ± 0.02d	0.85 ± 0.02d	0.75 ± 0.09d	0.38 ± 0.02d
	HRW+ZnPPIX→PEG	1.11 ± 0.03d	0.87 ± 0.10cd	0.76 ± 0.09cd	0.39 ± 0.02d
	CO+Hb→PEG	1.15 ± 0.08c	0.84 ± 0.04e	0.79 ± 0.02c	0.43 ± 0.05cd
	CO+ZnPPIX→PEG	1.15 ± 0.05c	0.86 ± 0.15d	0.77 ± 0.06cd	0.46 ± 0.07c
Chl b	Con→Con	0.48 ± 0.01a	0.47 ± 0.04ab	0.47 ± 0.03ab	0.45 ± 0.03ab
	Con→PEG	0.47 ± 0.02a	0.30 ± 0.03b	0.25 ± 0.02c	0.13 ± 0.02c
	HRW→PEG	0.48 ± 0.01a	0.44 ± 0.04a	0.37 ± 0.02b	0.34 ± 0.03b
	CO→PEG	0.49 ± 0.03a	0.47 ± 0.03ab	0.47 ± 0.01ab	0.45 ± 0.02a
	CO+HRW→PEG	0.51 ± 0.05a	0.49 ± 0.02a	0.48 ± 0.04a	0.46 ± 0.03a
	HRW+Hb→PEG	0.47 ± 0.02a	0.29 ± 0.02c	0.25 ± 0.01c	0.12 ± 0.02c
	HRW+ZnPPIX→PEG	0.47 ± 0.05a	0.30 ± 0.05b	0.24 ± 0.01d	0.12 ± 0.02c
	CO+Hb→PEG	0.46 ± 0.02a	0.30 ± 0.05b	0.25 ± 0.05c	0.12 ± 0.05c
	CO+ZnPPIX→PEG	0.47 ± 0.04a	0.31 ± 0.03b	0.25 ± 0.03c	0.11 ± 0.06d
Chla+b	Con→Con	1.62 ± 0.10bc	1.58 ± 0.03a	1.63 ± 0.07a	1.61 ± 0.10a
	Con→PEG	1.61 ± 0.04bc	1.21 ± 0.09b	1.04 ± 0.11c	0.54 ± 0.02c
	HRW→PEG	1.66 ± 0.02b	1.53 ± 0.01a	1.31 ± 0.03b	1.28 ± 0.06b
	CO→PEG	1.75 ± 0.04a	1.58 ± 0.01a	1.64 ± 0.02a	1.64 ± 0.03a
	CO+HRW→PEG	1.81 ± 0.01a	1.59 ± 0.03a	1.63 ± 0.04a	1.61 ± 0.05a
	HRW+Hb→PEG	1.55 ± 0.04c	1.14 ± 0.04d	0.99 ± 0.01d	0.50 ± 0.03c
	HRW+ZnPPIX→PEG	1.59 ± 0.04bc	1.17 ± 0.10cb	1.00 ± 0.10c	0.51 ± 0.04c
	CO+Hb→PEG	1.55 ± 0.05c	1.19 ± 0.02c	1.01 ± 0.03c	0.50 ± 0.05c
	CO+ZnPPIX→PEG	1.58 ± 0.02bc	1.21 ± 0.05b	0.99 ± 0.01d	0.50 ± 0.03c

*Explants were pretreated with or without 50% (v/v) HRW or 30% (v/v) CO aqueous solution for 48 h, and then transferred on agar plate containing 0 or 0.3% PEG. Explants without PEG treatments were used as the control (Con→Con). Chlorophyll content was measured at 0, 12, 24, and 48 h, respectively. Means and SE values were calculated from at least three independent experiment (*n* = 3). Treatment means with the same letter within the same column are not significantly different at *P* < 0.05 according to Duncan’s multiple range tests.*

**FIGURE 4:**
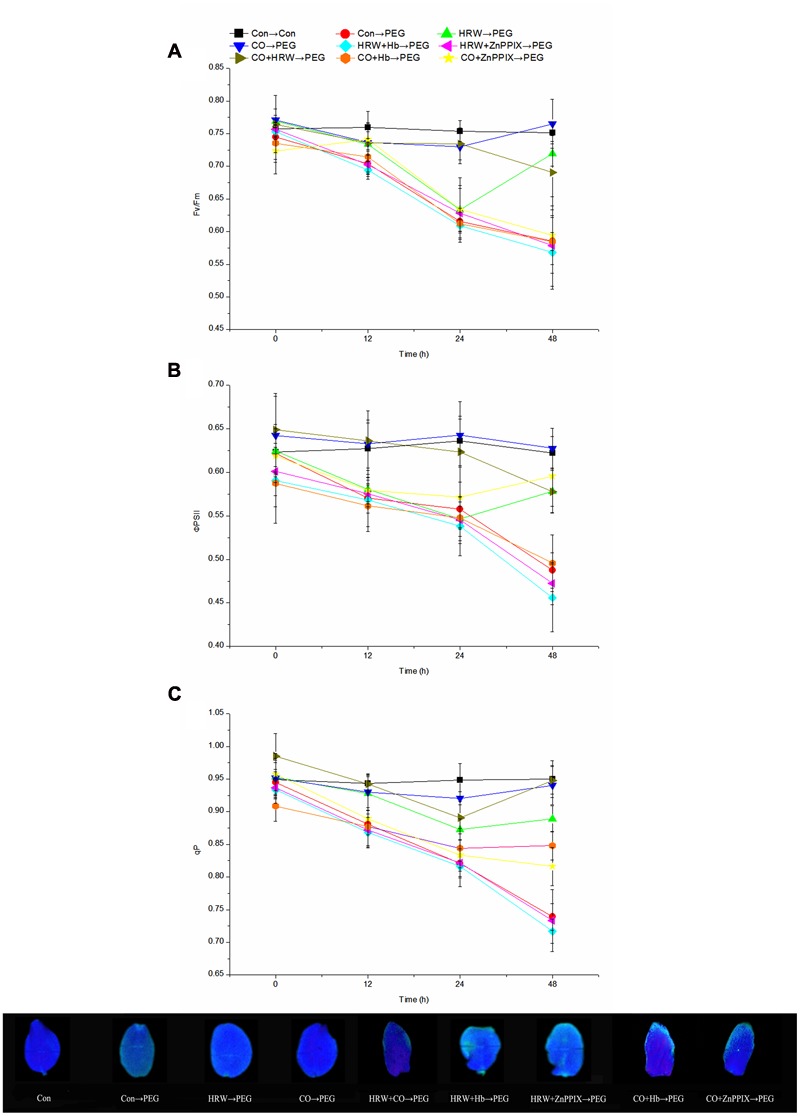
**Effects of H_2_ and CO on chlorophyll fluorescence parameters in the cotyledon of cucumber explant during the adventitious rooting process.** Rooting were Con (square), PEG (circle), CO (regular triangle), HRW (inverted triangle), CO+HRW (diamond), HRW+Hb (left triangle), HRW+ZnPPIX (right triangle), CO+Hb (hexagon), or CO+ZnPPIX (pentagram) as indicated. The concentrations of PEG, HRW, and CO were used at 0.3% (w/v), 50% (v/v), and 500 μM, respectively. Fv/Fm **(A)**, ΦPS II **(B)**, and qP **(C)** were determined after 0, 12, 24, 48 h of PEG treatment. Vertical bars represent mean ± SE value from three independent experiments.

The effects of H_2_ and CO on chlorophyll fluorescence parameters were studied during adventitious rooting under drought stress. During the experiment period, there was a continued decrease for all chlorophyll fluorescence parameters in PEG, HRW/CO+Hb and HRW/CO+ZnPPIX treatments (**Figure 4**). These parameters in HRW treatment reached minimum at 24 h and then increased until the end. When the cucumber explants were transferred to PEG treatment, the *F*v/*F*m decreased at 12, 24, and 48 h. However, HRW, CO, and CO+HRW treatments increased the *F*v/*F*m at 12, 24, and 48 h compared with PEG treatment. Furthermore, when the Hb or ZnPPIX was added to HRW/CO, the positive effect of HRW/CO on the parameters was significantly reversed (*P* < 0.05; **Figure 4A**).

Compared with the control, PEG treatment significantly reduced the ΦPSII at 12, 24, and 48 h (**Figure 4B**). In addition, treatments with HRW, CO, and CO+HRW yielded significantly higher ΦPSII values than PEG treatment (*P* < 0.05). CO treatment yielded maximum values among all treatments during the period. Meanwhile, the explants treated with HRW/CO+Hb and HRW/CO+ZnPPIX showed lower ΦPSII than those treated with HRW/CO (**Figure 4B**).

**Figure 4C** showed that the tendency of qP changed almost concurrently with that of *F*v/*F*m. It appeared that HRW, CO, and CO+HRW could alleviate the negative effect of PEG on qP. However, the addition of Hb or ZnPPIX in HRW/CO suppressed the stimulating effect of HRW/CO on the qP under drought condition. For example, the qP in HRW+Hb and HRW+ZnPPIX treatments at 48 h were 19.46 and 17.54% lower than that in HRW treatment, respectively; the qP in CO+Hb and CO+ZnPPIX treatments at 48 h were 9.85 and 13.23% lower than that in CO treatment, respectively. These results suggest that both H_2_ and CO affected chlorophyll fluorescence parameters during adventitious rooting under drought stress.

### Effect of Pretreatments with H_2_ and CO on the Levels of TBARS, O_2_^-^, and H_2_O_2_ during Adventitious Rooting under Simulated Drought Stress

**FIGURE 5:**
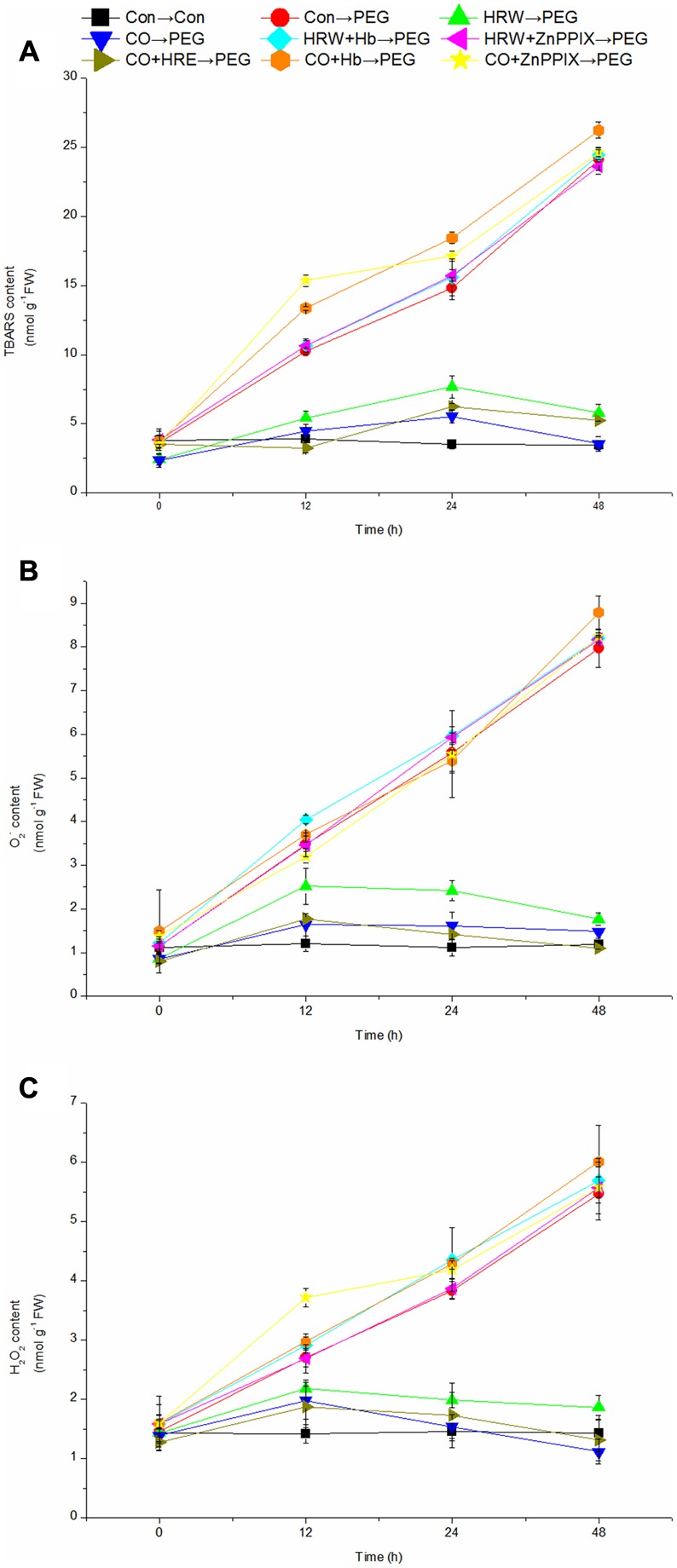
**Effects of H_2_ and CO on the levels of TBARS, O_2_^-^, and H_2_O_2_ in the adventitious root of cucumber during the rooting process.** Rooting were Con (square), PEG (circle), CO (regular triangle), HRW (inverted triangle), CO+HRW (diamond), HRW+Hb (left triangle), HRW+ZnPPIX (right triangle), CO+Hb (hexagon), or CO+ZnPPIX (pentagram) as indicated. The concentrations of PEG, HRW, and CO were used at 0.3% (w/v), 50% (v/v), and 500 μM, respectively. The content of TBARS **(A)**, O_2_^-^**(B)**, and H_2_O_2_ **(C)** was determined after 0, 12, 24, 48 h of PEG treatment. Vertical bars represent mean ± SE value from three independent experiments.

As shown in **Figure 5**, the levels of TBARS, O_2_^-^, and H_2_O_2_ in the control explants remained constant during the period. The levels of TBARS, O_2_^-^, and H_2_O_2_ in PEG, HRW/CO+Hb, and HRW/CO+ZnPPIX treatments continuously increased to the end of the experiment. In addition, **Figure 5A** showed that the TBARS level in HRW, CO, and CO+HRW treatments reached the highest at 24 h and then decreased. Whereas the levels of O_2_^-^ and H_2_O_2_ in HRW, CO, and CO+HRW treatments increased during 0–12 h, and then decreased after 12 h (**Figures 5B,C**). Compared with the control, PEG treatment caused 162.77, 320.94, and 592.38% increase in TBARS level at 12, 24, and 48 h, respectively (**Figure 5A**). When explants were transferred to PEG treatment, HRW-, CO-, and CO+HRW-pretreated explants had reduced TBARS levels at 12, 24, and 48 h compared with PEG treatment. However, HRW/CO+Hb- and HRW/CO+ZnPPIX-pretreated explants had higher TBARS levels than only HRW/CO- and CO+HRW-pretreated explants at 12, 24, and 48 h (**Figure 5A**).

PEG treatment resulted in significant higher levels of O_2_^-^ and H_2_O_2_ than those in the control at 12, 24, and 48h (**Figures 5B,C**). Meanwhile, HRW, CO, and CO+HRW treatments significantly decreased the levels of O_2_^-^ and H_2_O_2_ in cucumber explants, compared with PEG treatment. When Hb and ZnPPIX were added into HRW/CO treatment, the effects of HRW/CO on the levels of O_2_^-^ and H_2_O_2_ were reversed (**Figures 5B,C**). Therefore, CO might be involved in H_2_-adjusted TBARS, O_2_^-^, and H_2_O_2_ content during adventitious rooting under drought stress.

### Effect of Pretreatments with H_2_ and CO on Activities of Antioxidant Enzymes during Rooting under Simulated Drought Stress

**FIGURE 6:**
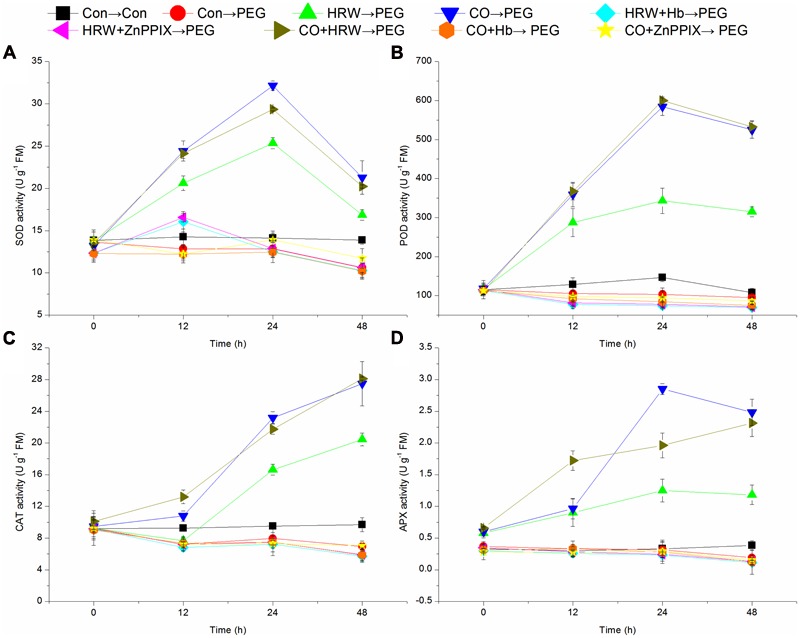
**Changes of antioxidant enzymes in cucumber explants during the rooting process.** Rooting were Con (square), PEG (circle), CO (regular triangle), HRW (inverted triangle), CO+HRW (diamond), HRW+Hb (left triangle), HRW+ZnPPIX (right triangle), CO+Hb (hexagon), or CO+ZnPPIX (pentagram) as indicated. The concentrations of PEG, HRW, and CO were used at 0.3% (w/v), 50% (v/v), and 500 μM, respectively. The activity of SOD **(A)**, POD **(B)**, CAT **(C)**, and APX **(D)** was determined after 0, 12, 24, 48 h of PEG treatment. Vertical bars represent mean ± SE value from three independent experiments.

As shown in **Figures 6A,B**, the activities of SOD and POD in the control treatment remained constant throughout the 48 h period of the experiment. Under drought condition, these activities in HRW-, CO-, and CO+HRW-pretreated explants rapidly increased at 0–24 h, followed by a gradual decrease until 48 h. The activities of SOD and POD in PEG treatment were slightly decreased within 12–48 h, whereas they significantly decreased in HRW/CO+Hb- and HRW/CO+ZnPPIX-pretreated explants during 0–48 h (*P* < 0.05). The activities of SOD and POD in PEG treatment were significantly lower than those in the control (*P* < 0.05; **Figures 6A,B**). The activities of SOD and POD in explants pretreated with HRW, CO, and CO+HRW were significantly higher than those in explants treated with PEG (*P* < 0.05). For example, POD activity in HRW treatment was 2.86-, 3.09-, and 3.27-fold increases over those in PEG treatment at 12, 24, and 48 h, respectively. Meanwhile, the activities of SOD and POD in HRW/CO+Hb- or HRW/CO+ZnPPIX-pretreated explants were lower than those in HRW/CO- pretreated explants (**Figures 6A,B**).

The CAT activity remained constant in the control within 0–48 h (**Figure 6C**). Under drought stress, the CAT activity of explants pretreated with HRW decreased during 0–12 h, and then increased sharply at 12–48 h. However, the CAT activity in explants pretreated with CO, HRW, and CO+HRW markedly increased during 0–48 h (*P* < 0.05). The CAT activity in explants pretreated with PEG, HRW/CO+Hb, and HRW/CO+ZnPPIX was slightly increased from 12 to 24 h and then decreased until 48 h. The CAT activity of in PEG treatment was 22.03, 16.31, and 28.45% lower than that in the control at 12, 24, and 48 h, respectively. The CAT activity in explants pretreated with HRW, CO, and CO+HRW was significantly higher than that in explants treated with PEG (*P* < 0.05). The CAT activity in the HRW+Hb, HRW+ZnPPIX, CO+Hb, and CO+ZnPPIX treatments was 72.12, 71.24, 78.66, and 74.35% lower than that in HRW treatment at 48 h, respectively (**Figure 6C**).

As shown in **Figure 6D**, the APX activity in control treatment remained constant within 0–48 h. When explants were transferred to PEG treatment, the activity increased within 0–24 h and then slightly decreased within 24–48 h in HRW, CO, and CO+HRW pretreatments. In PEG, HRW/CO+Hb and HRW/CO+ZnPPIX treatments, the APX activity continuously decreased within 0–48 h. The activity of APX in PEG treatment was 6.06 and 50.00% lower than that in the control at 24 and 48 h, respectively. HRW, CO, and CO+HRW treatments resulted in significantly higher APX activity than PEG treatment (*P* < 0.05). For example, when compared with PEG treatment, the APX activity in HRW, CO, and CO+HRW treatments increased by 303.22, 819.35, and 519.72% at 24 h, respectively. The APX activity in HRW/CO+Hb or HRW/CO+ZnPPIX treatments was significantly lower than that in HRW treatment (*P* < 0.05; **Figure 6D**). Therefore, CO was involved in H_2_-induced enhancement of the SOD, POD, CAT, and APX activities during adventitious rooting under drought condition.

### Effects of Pretreatments with H_2_ and CO on Levels of Osmotic Regulators during Adventitious Rooting under Simulated Drought Stress

**FIGURE 7:**
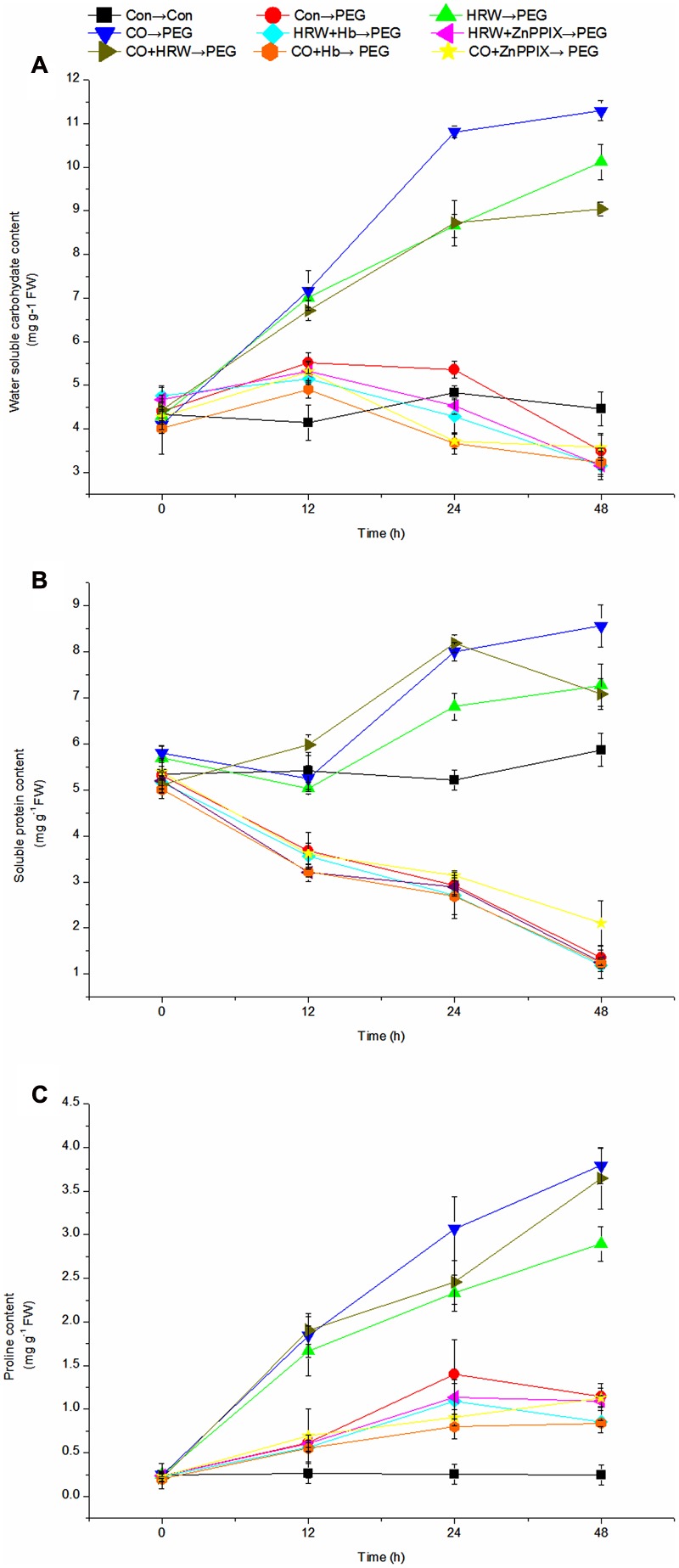
**Changes of osmotic regulator levels in cucumber explants during the rooting process.** Rooting were Con (square), PEG (circle), CO (regular triangle), HRW (inverted triangle), CO+HRW (diamond), HRW+Hb (left triangle), HRW+ZnPPIX (right triangle), CO+Hb (hexagon), or CO+ZnPPIX (pentagram) as indicated. The concentrations of PEG, HRW, and CO were used at 0.3% (w/v), 50% (v/v), and 500 μM, respectively. The content of water soluble carbohydrate **(A)**, soluble protein **(B)**, and proline **(C)** was determined after 0, 12, 24, 48 h of PEG treatment. Vertical bars represent mean ± SE value from three independent experiments.

As showed in **Figure 7A**, the level of water soluble carbohydrate in the control slightly increased within 12–24 h, and then decreased within 24–48 h. Under drought stress, the water soluble carbohydrate content increased continuously in HRW, CO, and CO+HRW treatments during 0–48 h. However, it decreased in HRW/CO+Hb, HRW/CO+ZnPPIX, and PEG treatments from 12 to 48 h. In addition, HRW, CO, and CO+HRW treatments caused a significant increase in water soluble carbohydrate content compare with PEG treatment (*P* < 0.05). Simultaneously, HRW/CO+Hb and HRW/CO+ZnPPIX treatments suppressed the positive effect of HRW/CO treatments. The water soluble carbohydrate content in the HRW+Hb, HRW+ZnPPIX, CO+Hb, and CO+ZnPPIX treatments was 68.87, 68.77, 71.42, and 68.39% lower than that in HRW treatment at 48 h, respectively (**Figure 7A**).

As shown in **Figure 7B**, the soluble protein content in the control decreased during 12–24 h, and then increased until 48 h. Under drought condition, the soluble protein content increased continuously in HRW and CO pretreatments during 12–48 h. The soluble protein content in CO+HRW treatment increased during 0–24 h, and then decreased until 48 h. In addition, the soluble protein content showed continued decrease in HRW/CO+Hb, HRW/CO+ZnPPIX, and PEG treatments during 0–48 h. Compared with the control, PEG treatment caused 31.85, 43.76, and 76.96% decrease in soluble protein content at 12, 24, and 48 h, respectively. Compared with the PEG treatment, HRW, CO, and CO+HRW treatments resulted in significantly higher soluble protein content (*P* < 0.05). However, if Hb or ZnPPIX were added to HRW/CO, the positive effect of HRW/CO was reversed (**Figure 7B**).

The proline content remains constant during the study period (**Figure 7C**). Its content increased continuously in HRW, CO, and CO+HRW treatments during 0–48 h. In addition, the proline content in PEG, HRW/CO+Hb, and HRW+ZnPPIX treatments increased during 0–24 h, and then decreased during 24–48 h. The proline content continuously increased during 0–48 h in CO+ZnPPIX treatment. Compared with the control, a higher content of proline in PEG treatment was obtained. As showed in **Figure 7C**, pretreatments with HRW, CO and CO+HRW produced a significant increase in proline content compared with PEG treatment (*P* < 0.05). Compared with PEG treatment, HRW, CO, and CO+HRW treatments resulted in 2.53-, 3.32-, and 3.19-fold proline content at 48 h, respectively. When Hb or ZnPPIX were added to HRW/CO, it resulted in a significant reduction in proline content (**Figure 7C**). Therefore, CO could be involved in H_2_-induced accumulation of water soluble carbohydrate, soluble protein, and proline during adventitious rooting in cucumber under drought stress.

## Discussion

Drought stress is one of the main abiotic stresses that affects plant growth and development. Drought stress affects growth, water and nutrient relations, photosynthesis, assimilate partitioning, and respiration in plants (Farooq et al., 2009). Thus, studying the mechanism of plant drought tolerance can provide a theoretical basis for improved crop production in the future.

Carbon monoxide was regarded as a cell protection mechanism against abiotic-induced damage (Yannarelli et al., 2006; Liu et al., 2010; Zhang et al., 2012). For example, Zheng et al. (2011) also demonstrated that CO could alleviate the oxidative damage effects of heavy metals on plants. In the present study, our results indicated that the applications of both low concentration of the CO donor Hemin (10, 100, and 500 μM, w/v) and CO aqueous solution (10, 30, and 50%, v/v) promoted adventitious rooting in cucumber explants under drought stress. However, high concentration of Hemin (1000 μM, w/v) and CO aqueous solution (50 and 100%, v/v) inhibited adventitious rooting under drought condition (**Figure 1**). The results are consistent with the findings of Dekker and Hargrove (2002), which showed that low concentration of CO (0.1%) in the air stimulated seed germination, whereas high concentration (75%) inhibited germination. Previous studies showed that CO exhibited positive effects on regulating plant root development in cucumber (Xuan et al., 2008). Xu et al. (2006) reported that the application of CO to mung bean hypocotyl induced *de novo* adventitious root formation in dose- and time-dependent manners. Our results indicate that CO was not only involved in drought stress responses but also required for adventitious root development in cucumber. Thus, CO could alleviate various abiotics tresses during the plant adventitious rooting.

H_2_ had an obvious promotion effect on adventitious rooting under drought stress. Some results from previous research by others indicated that H_2_ had great potential applications within plant growth (Jin et al., 2013; Cui et al., 2014). H_2_ was found to enhance salt tolerance in *Arabidopsis* (Xie et al., 2012) and drought tolerance in *Arabidopsis* (Xie et al., 2014) and in *M. sativa* (Jin et al., 2016). Recent studies revealed that H_2_ was involved in alleviating ultraviolet A and ultraviolet B irradiation in two varieties of radish sprouts and in *M*. *sativa* (Su et al., 2014; Xie et al., 2015). Our results were consistent with the results of Wu et al. (2015), who found that H_2_ promoted root growth in Chinese cabbage under Cd stress. Xie et al. (2012) reported that a high concentration of HRW had a negative effect on the primary root growth in *Arabidopsis* seedlings under salinity stress. These findings are indications that suitable concentration of H_2_ has important functional implications in promoting growth and development of plants under abiotic stresses.

Studies have shown that H_2_ signal transduction pathways do not always work dependently. Zhu et al. (2016b) demonstrated that NO is required for H_2_-induced adventitious rooting in cucumber. CO alleviated osmotic-induced wheat seed germination inhibition and lipid peroxidation which required the participation of NO (Liu et al., 2010). Furthermore, H_2_ was involved in phytohormone and stress responses in plants (Zeng et al., 2013). A functional interaction of CO and phytohormone has been demonstrated in regulating plant growth and development (Wang and Liao, 2016). Jin et al. (2013) found that H_2_O_2_ and heme oxygenase-1 were involved in H_2_-induced osmotic stress tolerance in alfalfa. She and Song (2008) illustrated that CO-induced stomatal closure probably was mediated by H_2_O_2_ signaling pathways in *V. Faba*. These results indicated that both the crosstalk among H_2_, CO, and other signal molecular optimized plants growth and enhanced the tolerance to various abiotic stresses. In addition, previous reports have shown that H_2_ may act synergistically with CO to enhance adventitious root development in non-stress conditions (Lin et al., 2014). In our study, we found that HRW, CO, and CO+HRW induced adventitious rooting under drought stress. Previous research demonstrated the cucumber produced CO at levels of 10 to 25 mL L^-1^ (Siegel et al., 1962). As a pharmacology method, ZnPPIX as a specific inhibitor of CO and Hb as CO scavenger were used to inhibit endogenous CO in H_2_/CO-treated explants. Interestingly, the results showed that the addition of Hb or ZnPPIX into H_2_/CO significantly reversed the positive effect of H_2_/CO. These results proved that CO was involved in H_2_-induced adventitious rooting under drought stress.

Previous study suggested that PEG treatment might reduce oxygen availability in plants (Chazen et al., 1995). In the study, PEG was used to simulate drought stress. Our results showed that PEG treatment significantly decreased leaf RWC during adventitious rooting, indicating that PEG treatment resulted in simulated drought stress. Addition of H_2_/CO significantly increased RWC during rooting under drought stress, resulting in higher adventitious root number. CO could regulate water balance and enhance salt tolerance in wheat seedling roots (Xie et al., 2008); HRW treatment also promoted RWC in *M. sativa* and improved its drought tolerance (Jin et al., 2016). Our results also indicated that H_2_/CO might promote adventitious rooting under drought stress through maintaining water holding capacity of plants. Moreover, Hb/ZnPPIX reversed the effects of H_2_/CO on RWC, suggesting CO is involved in water balance induced by H_2_ during adventitious root development under drought stress.

Our results suggest that HRW, CO, and CO+HRW significantly increased the contents of chl a, chl b, and chl a+b. Previous studies showed that H_2_ increased chlorophyll content and alleviated ultraviolet-B-triggered oxidative damage in *M. sativa* (Xie et al., 2015). Moreover, the application of CO increased chlorophyll content in wheat leaf and promoted wheat seed germination under drought stress (Liu et al., 2010). Liao et al. (2012) demonstrated that the enhanced chlorophyll content could effectively mitigate the adverse effect of drought stress in marigold and promoted adventitious rooting. Therefore, H_2_ and CO enhanced leaf chlorophyll content and then increased photosynthesis during drought and promoted adventitious rooting. In our study, drought stress led to the decrease in *F*v/*F*m, ΦPSII and qP. However, HRW, CO, and CO+HRW alleviated the negative effect of drought on *F*v/*F*m, ΦPSII, and qP. The result was consistent with the findings of Zhang et al. (2015) who reported that H_2_ enhanced Fv/Fm, ΦPSII and qP in high light stress in maize. Zhang et al. (2012) reported that CO induced advantageous effects on the attenuation of salt-stress inhibition in *Cassia obtusifolia* seeds and seedlings. Our results indicated that HRW, CO, and CO+HRW induced increase in *F*v/*F*m, ΦPSII, and qP, and promoted adventitious rooting under drought condition. In our experiment, when Hb or ZnPPIX was added to HRW/CO, the positive effect of HRW/CO was reversed. This demonstrates that CO might be involved in H_2_-regulated chlorophyll content and chlorophyll fluorescence parameters during adventitious rooting under drought stress.

The levels of TBARS, O_2_^-^, and H_2_O_2_ could reflect the degree of oxidative damage to membrane lipids. In the present study, the TBARS level was significantly increased under drought stress. However, the TBARS levels were significantly decreased in HRW, CO, and CO+HRW treatments. Higher lipid peroxidation has also been reported in salt stress-sensitive rice varieties (Dionisio-Sese and Tobita, 1998). Cui et al. (2013) reported that the addition of HRW at 10% saturation significantly decreased contents of TBARS, and inhibited the appearance of Cd toxicity symptoms in alfalfa plants. Drought stress remarkably increased the TBARS level, but CO significantly decreased the TBARS content in plants subjected to salinity stress (Huang et al., 2006). Thus, it showed that H_2_ and CO could alleviate lipid peroxidation caused by drought stress. Also, our results suggested that drought stress resulted in a significant increase in the levels of O_2_^-^ and H_2_O_2_, and HRW, CO, and CO+HRW could reverse it, showing that the drought stress-induced damage to cucumber explants had been alleviated. Jin et al. (2013) made a similar observation and reported that alfalfa seedlings pretreated with 0.39 mM H_2_ exhibited remarkable alleviation of oxidative damage. The exposure of alfalfa seedlings to HgCl_2_ triggered the production of reactive oxygen species (ROS), stunted growth and increased lipid peroxidation. However, such negative effects were obviously blocked by H_2_ (Cui et al., 2014). In another study, CO significantly decreased the contents of O_2_^-^ and H_2_O_2_ in Indian mustard under Hg stress (Meng et al., 2011). Our experiment revealed that HRW, CO, and CO+HRW enhanced drought tolerance in cucumber explants by decreasing TBARS, O_2_^-^, and H_2_O_2_ content. Moreover, when Hb or ZnPPIX were added to HRW/CO pretreatments, the positive effects of HRW/CO were reversed. Under Cu toxicity, the increased lipid peroxidation and contents of ROS had previously been reported in the mountain ginseng adventitious roots (Rajesh et al., 2008). These results suggested that H_2_ and CO could effectively mitigate the damage of drought stress via decreasing the levels of TBARS, O_2_^-^, and H_2_O_2_ during adventitious rooting under drought condition.

Plants possess an efficient antioxidant system to adjust to oxidative damage from biotic and abiotic stresses. Upon exposure to abiotic stresses, tolerant cells activate their antioxidant enzymatic system, which starts quenching the ROS and then protecting the cell (Wang and Liao, 2016). We found that the activities of SOD, POD, CAT, and APX were reduced under drought stress. HRW, CO, and CO+HRW treatments relieved the decrease of antioxidant enzymatic under drought stress. However, HRW, CO, and CO+HRW treatments increased the activities of SOD, POD, CAT, and APX in cucumber adventitious rooting under drought stress. Studies have showed that H_2_ alleviated salinity stress in rice, which is associated with the induction of SOD, CAT, and APX (Xu et al., 2013). Cui et al. (2013) observed that H_2_ exhibited beneficial effects on the activities of SOD, POD, and APX and enhanced Cd stress tolerance in *M. sativa*. Moreover, Hemin and CO aqueous solution dose-dependently enhanced the activities of CAT and SOD, and alleviated the inhibition of seed germination and seedling growth under salt stress (Liu et al., 2007). CO increased the activities of SOD, POD, CAT, and APX, and it alleviated the inhibition of seed germination in *C. obtusifolia* L. and seedling growth under salinity stress (Zhang et al., 2012). Also, we found that H_2_ and CO improved drought tolerance in cucumber largely by increasing activities of SOD, POD, CAT, and APX. Meanwhile, Hb and ZnPPIX inhibited the promoted effects of HRW/CO under drought stress. The increase of POD activity has been known to be a rooting signal during root primordium formation (Ann-Caroline et al., 1991). There is a close relationship between the antioxidant enzymes and the formation of adventitious roots (Liao et al., 2010). The evidence from our study supported the hypothesis that under our experimental conditions, H_2_ and CO promoted adventitious root development under drought stress, and CO was involved in H_2_-inducedadventitious rooting under drought stress.

The changes in water soluble carbohydrate content may be linked to external environment. In the present study, we found that drought resulted in decreased content of water soluble carbohydrate. However, the application of HRW, CO, and CO+HRW treatments to cucumber explants subjected to drought stress increased water soluble carbohydrate content. Indeed, CO was able to promote the activities of amylase and induced the formation of water soluble carbohydrate, thus resulting in the alleviation of oxidative damage caused by salt stress in *Oryza sativa* (Liu et al., 2007). Previous study showed that Hemin and CO enhanced the contents of total water soluble carbohydrate, soluble protein, and proline in *C. obtusifolia* L. leaf under salinity stress, which are contribute in improving plants resistance to stress (Zhang et al., 2012). Together, these results suggested that H_2_ and CO inhibited the decrease in water soluble carbohydrate content and subsequently promoted rooting under drought stress. Our study also showed that the soluble protein and proline accelerated the decline during rooting under drought condition. Soluble protein content was affected in tomato exposed to salt stress (Zeynep et al., 2010). CO modulated the accumulation of proline and increased tolerance in Indian mustard in Hg stress (Meng et al., 2011). In our experiment HRW, CO, and CO+HRW treatments enhanced soluble protein and proline content. The levels of water soluble carbohydrate, soluble protein, and proline should be important considerations in the study of adventitious root development (Klerk, 1996). According to Zavattieri et al. (2009), the application of exogenous carbon sources (sucrose or glucose) was beneficial for the rooting of microshoots of *Pinus pinea*. Maintaining the levels of soluble carbohydrate and protein promoted adventitious rooting in marigold explants (Liao et al., 2012). Similarly, our results confirmed the importance of CO in H_2_-induced adventitious root development under drought stress. Thus, the results have an important functional implication, expanding and enriching the possibilities for H_2_ and CO during adventitious root development under various stresses.

The present study provides new insights into the roles and interactions of H_2_ and CO in their regulation of adventitious root development in cucumber seedlings under drought stress. We, therefore, conclude that H_2_ and CO the development of adventitious roots in cucumber seedlings by alleviating the negative effects of drought on RWC, chlorophyll content, chlorophyll fluorescence, antioxidant systems and osmotic strength, and inhibit oxidative damage of cucumber explants. Concurrently, the positive effect of HRW/CO was reversed by Hb or ZnPPIX, indicating that CO was involved in H_2_-induced adventitious root development in cucumber seedlings under drought stress.

## Author Contributions

WL designed the experiments; YC, MW and WL performed the experiments; YC, LH, MW and CL performed data analysis; YC and LH wrote the manuscript; WL and MD edited the manuscript.

## Conflict of Interest Statement

The authors declare that the research was conducted in the absence of any commercial or financial relationships that could be construed as a potential conflict of interest.
